# Managing norovirus outbreaks: opportunities for organisational learning

**DOI:** 10.1186/1753-6561-5-S6-P99

**Published:** 2011-06-29

**Authors:** EJ Murray, R Edwards, A Holmes

**Affiliations:** 1 Department of Infectious Diseases and Immunity, Imperial College London, London, UK; 2 Centre for Infection Prevention and Management, Imperial College London, London, UK

## Introduction / objectives

This study examines the impact of behavioural factors in the successful management of an organisation-wide Norovirus outbreak in a UK hospital.

## Methods

A retrospective qualitative content analysis of a Norovirus outbreak was undertaken, using critical incident analysis as the basis for conducting semi-structured interviews with 24 key clinical and managerial participants. Behavioural references were mapped to a behavioural framework (Weick and Sutcliffe, 2007) that describes positive behaviours, known as ‘mindful behaviours’ and negative behaviours, known as ‘mindless behaviours’ which contribute to high and low performance respectively in the context of an unexpected event, using a qualitative software tool (NVivo). The resulting data was analysed using frequency tables.

## Results

The results demonstrated a higher count of mindful behaviours than mindless behaviours in what participants perceived to be a successfully managed outbreak. Mindful behaviours included the engagement of clinical experts, daily reviews of hospital capacity and capability, prioritisation of patient safety over parochial interests, rigorous implementation of clinical reviews and effective communication with clinicians. Successful management was defined by participants as the appropriate balance between safety and performance that: minimised morbidity, ensured the hospital continued to meet its performance targets and maintained the hospital’s reputation externally.

## Conclusion

This study has an in-depth focus on the behaviours that facilitate and hinder the successful management of a potentially highly disruptive Norovirus outbreak. The lessons learnt can contribute to improved management of future outbreaks and an enhanced understanding of the behavioural drivers of an effective outbreak response.

## Disclosure of interest

None declared.

